# Case of Hœmaturia—Death—Carcinoma of Bladder

**Published:** 1848

**Authors:** Austin Flint

**Affiliations:** Professor of Principles and Practice of Medicine in the University of Buffalo


### ARTICLE III.

*Case of Hæmaturia—Death—Carcinoma of Bladder.* By Austin Flint, M. D., Professor of Principles and Practice of Medicine in the University of Buffalo.

The following case, which came under observation a few weeks since, is reported from brief notes made at the time of its occurrence: G. J. P., aged about 60; had generally enjoyed good health, but of late had presented a pallid aspect, and *had complained frequently, more especially at night, of sharp cutting pains in the lower part of the abdomen;* applied on the 4th ult. for advice, stating that for two or three days his urine had been bloody, and that he was then troubled with some dysury. On examination of the urine, made shortly afterward, it appeared to contain considerable blood. On the evening of the same day, being requested to visit him, I found him in great distress, and unable to void urine except in very small quantity. He succeeded, however, by his own efforts, in relieving the bladder shortly after I entered the room. An enema of starch and tinct. opii was directed, should the dysury return.

On the 5th, found he had suffered much from strangury during the night. The anodyne enema had not been administered until morning. Was now sleeping, and the urine had escaped abundantly during his sleep. The bed appeared deluged with blood. He suffered much through the day from strangury, and the urine continued to be very bloody. Prescribed infus. uvæ ursi, copaiva mixture, laudanum enemas frequently repeated, and hip baths. There was no pain, nor tenderness in loins, but sensitiveness to pressure over hypogastric region. Up to the 13th he continued to suffer greatly from difficult micturition, the bladder, however, relieving itself, for the most part, under the influence of anodyne enemas, which were repeated at short intervals, and of hip baths, or fomentations. The quantity of blood gradually diminished, and the urine assumed nearly its natural appearance. The copaiva mixture, after a couple of days, was omitted, and the tinct. mur. ferri freely given. The uvæ ursi and mucilages were continued. There was no febrile movement, and his muscular strength was not much impaired. On the 14th he passed a very distressed night, and the urine again became very bloody. Dr. A. S. Sprague saw the patient with me several times on this day. The pulse on this day became very feeble, so as scarcely to be felt; the skin was cold, and the muscular force much prostrated. On getting out of bed to make efforts to relieve the bladder, he fainted, and his family were obliged to call in assistance to remove him from the floor to the bed. On examination of the hypogastrium, the distended bladder could be readily felt, and, from its want of resiliency, it seemed to be filled with a solid mass. The suffering from dysuria was extreme. Dr. Sprague attempted to introduce a catheter, but on passing it a short distance, the instrument encountered an obstacle which could not be overcome, and the attempt occasioned excruciating pain. The treatment consisted in the free administration of opiates by the mouth, and per ano. He continued to experience great agony up to the time of his death, which occurred on the morning of the 15th.

On examination, after death, the bladder was found largely distended with coagula, and a fluid resembling bloody serum. The exact amount of contents in weight was not ascertained, but the coagula alone, as it was estimated, amounted to between two and three pounds. The amount of hemorrhage, it would seem, was sufficient to have caused death. The contents of the bladder exhibited no evidences of decomposition, showing that the effusion was recent. It probably occurred on the 13th, when bloody urine re-appeared, and the patient presented the alarming prostration mentioned in the history.

On removing the bladder, the lining membrane appeared thickened, but no traces of inflammation or vascular injection were apparent. At the inferior portion near the urethral orifice, was a circular patch nearly as large as a half dollar, of carcinomatous degeneration. It was somewhat elevated, perfectly white, and of a medullary consistence. No ecchymosis was apparent, nor could any orifice into a vessel be discovered, whence the blood might have escaped.

In the only case, in addition to the foregoing, of excessive hæmaturia which has fallen within the knowledge of the writer, there was found, on examination after death, malignant disease of the mucous membrane of the bladder.

*Buffalo Med. Jour.*

